# Transient absorption changes in vivo during photodynamic therapy with pulsed-laser light

**DOI:** 10.1038/sj.bjc.6690361

**Published:** 1999-05-01

**Authors:** B W Pogue, T Momma, H C Wu, T Hasan

**Affiliations:** Wellman Laboratories of Photomedicine, Department of Dermatology, and Department of Pathology, Massachusetts General Hospital, Boston, MA 02114, USA

**Keywords:** photosensitizer, BPD-MA, pulsed-laser, optical dosimetry, tumour

## Abstract

High intensity pulsed-laser light can be used to excite absorbing molecules to transient states in large proportions. The laser-induced spectral changes can be characterized by transient changes in light propagation; through the tissue provided the excited states of these molecules have altered absorption spectra. Characterization of these transient changes may then be used to exploit new mechanisms in photosensitization and/or to optimize photobiological effects. In this study, transmittance and reflectance were measured as a function of laser pulse energy, from tissue-simulating media as well as in rat muscle and liver slices, both with and without the photosensitizer benzoporphyrin derivative monoacid (BPD-MA) present. There was a transient decrease in absorption from the photosensitizer at peak pulse irradiance in the range of 100–1000 W cm^–2^. The depth of photodynamic treatment-induced tissue necrosis was measured in a subcutaneous prostate cancer model in Copenhagen rats. A comparison between continuous wave irradiation and pulsed irradiation with the same average incident irradiance showed no statistically significant difference in the depth of necrosis at 48 h after irradiation. These results indicate that photosensitizer population-state changes are measurable in tissues and may provide a method for measuring triplet-state properties of photosensitizer in vivo, but for BPD-MA at clinically used concentrations these changes do not significantly affect the depth of photodynamically-induced tissue damage. © 1999 Cancer Research Campaign


*British Journal of Cancer* (1999) **80**(3/4), 344–351
© 1999 Cancer Research Campaign
Article no. bjoc.1998.0361

### Transient absorption changes in vivo during photodynamic therapy with pulsed-laser light

**BW Pogue[1], T Momma[1], HC Wu[2] and T Hasan[1]**

[1]Wellman Laboratories of Photomedicine, Department of Dermatology, and [2]Department of Pathology, Massachusetts General Hospital, Boston, MA 02114, USA

**Summary** High intensity pulsed-laser light can be used to excite absorbing molecules to transient states in large proportions. The laser-induced spectral changes can be characterized by transient changes in light propagation; through the tissue provided the excited states of these molecules have altered absorption spectra. Characterization of these transient changes may then be used to exploit new mechanisms in photosensitization and/or to optimize photobiological effects. In this study, transmittance and reflectance were measured as a function of laser pulse energy, from tissue-simulating media as well as in rat muscle and liver slices, both with and without the photosensitizer benzoporphyrin derivative monoacid (BPD-MA) present. There was a transient decrease in absorption from the photosensitizer at peak pulse irradiance in the range of 100–1000 W $cm^{-2}$. The depth of photodynamic treatment-induced tissue necrosis was measured in a subcutaneous prostate cancer model in Copenhagen rats. A comparison between continuous wave irradiation and pulsed irradiation with the same average incident irradiance showed no statistically significant difference in the depth of necrosis at 48 h after irradiation. These results indicate that photosensitizer population-state changes are measurable in tissues and may provide a method for measuring triplet-state properties of photosensitizer in vivo, but for BPD-MA at clinically used concentrations these changes do not significantly affect the depth of photodynamically-induced tissue damage.

**Keywords:** photosensitizer; BPD-MA; pulsed-laser; optical dosimetry; tumour

Photodynamic therapy (PDT) is a treatment modality for a variety of diseases using a sensitizing agent (photosensitizer) activated by optical radiation (Pass, 1993; Hasan and Parrish, 1996). The photosensitizer can be applied topically or administered systemically and is somewhat preferentially accumulated in diseased tissues. This diseased area is then activated by light resonant with the absorption band of the ground-state sensitizer and a large fraction of the sensitizer molecules are excited to the first singlet-state. These excited sensitizer molecules can intersystem cross to the triplet-state and are highly quenched by ground-state (triplet-state) oxygen to produce singlet-state oxygen, which is generally believed to be the major cytotoxic species in PDT. These active species can cause direct cell death and destruction of blood vessels when produced in large numbers (usually $>10^{12}$ active molecules per cell), depending on the physical localization of the photosensitizer and the availability of light, photosensitizer and oxygen. While the number of applications for this treatment modality have been increasing in the last few years (Pass, 1993; Hasan and Parrish, 1996), its use has been mainly restricted to superficial tissue treatments, such as bladder wall carcinoma (Rosenberg and Williams, 1986; Jocham et al, 1990; D'Hallewin and Baert, 1995), palliation of oesophageal cancer (Narayan and Sivak, 1994; Moghissi et al, 1995), basal cell carcinoma (Wilson et al, 1992; Lui and Anderson, 1993) and as adjuvant treatment after surgical debulking of tumours (Muller and Wilson, 1990). A major limitation to treating larger tissue volumes is the high attenuation of light in tissue, causing the deposited photodynamic dose to be high at the incident surface and decreasing exponentially with depth.

More recent studies directed at increasing the depth of treatment have examined using second-generation photosensitizers for PDT which have absorption bands in the 650–700 nm range, where there is the least amount of optical scattering and absorption from the tissue (Gomer, 1991). The penetration of light in tissue can be maximized with wavelength manipulation to a limited extent only and is maximal between 650 and 900 nm, without a great variation in this region because of relatively weak changes in endogenous chromophore concentration (Svaasand et al, 1990). In addition, the depth of light penetration can be limited due to self-shielding of the light because of absorption from the photosensitizer in the tissue (Bown et al, 1986; Wilson et al, 1986) at relatively high concentrations and extinction coefficients of the photosensitizer. It has been hypothesized that high-intensity pulsed light could allow a deeper penetration of the treatment in tissue than continuous wave (cw) light by causing a transient decrease in the absorption of the photosensitizer during the time of the pulse. This process allows the latter half of the pulse to pass through the surface layers with less attenuation (Patterson and Wilson, 1994). There have been several studies in recent years examining the use of pulsed lasers in PDT, which can be divided roughly into either physical chemistry-based research (Andreone et al, 1982; Andreone, 1987; Keir et al, 1987; Shea et al, 1990; Smith et al, 1994; Stiel et al, 1993), or phenomenologic biologic studies (Cowled et al., 1984; McKenzie and Carruth, 1986; Ben-Hur et al, 1987; Ferrario et al, 1991; Shikowitz, 1992; Panjehpour et al, 1993; Rausch et al, 1993; Okunaka et al, 1992; Pe et al., 1994). Several in vivo experimental papers have compared pulsed peak powers in the range of

*Received 12 June 1998*
*Revised 4 November 1998*
*Accepted 9 November 1998*

*Correspondence to:* T Hasan

**Table 1** Depth of necrosis as measured from histologic sections 48 h after PDT

| Irradiation protocol | Depth of necrosis (s.d.) (mm) | Average (mm) |
|---|---|---|
| cw (50 J cm$^{-2}$) (200 mW cm$^{-2}$) | 10.1 (0.5), 8.2 (0.3), 12.5 (0.4), 5.1 (0.3), 10.4 (0.4), 8.9 (0.3), 5.4 (0.2) | 8.7 (2.5) |
| Pulsed (50 J cm$^{-2}$) (125 mW/cm$^{-2}$) | 7.5 (0.4), 5.5 (0.4), 8.4 (0.3), 7.6 (0.4), 11.3 (0.9), 9.9 (0.2), 8.1 (0.2), 3.2 (0.4) | 7.7 (2.3) |
| Pulsed (50J /cm$^{-2}$) (225 mW/cm$^{-2}$) | 9.9 (0.2), 12.1 (0.2), 8.1 (0.1), 6.4 (0.0), 8.5 (1.8), 8.3 (0.2), 7.0 (0.2), 8.0 (0.2) | |

100–1000 W cm$^{-2}$ to cw irradiation with no significant difference in tissue treatment depth and no difference in clinical outcome for PDT (McKenzie and Carruth, 1986; Ferrario et al, 1991; Shikowitz, 1992; Panjehpour et al, 1993), yet studies with peak pulse powers above $10^5$ W cm$^{-1}$ have demonstrated increases in the PDT-affected tumour response rate (Rausch et al, 1993) and increase in the depth of necrosis (Okunaka et al, 1992) with pulsed-laser irradiation as compared to cw irradiation in PDT. Unfortunately, there is no definitive explanation for these improvements in PDT response with high-intensity irradiation, nor is there any direct evidence that there are transient absorption changes in vivo which are associated with photosensitizer population changes. In an attempt to better understand the effect of high-intensity pulsed irradiation for PDT, we report here the first study which (i) experimentally tests the hypothesis that at moderate concentrations of the photosensitizer benzoporphyrin derivative (BPD-MA), high-intensity irradiation can lead to alterations in the propagation of light at 690 nm compared to cw irradiation; (ii) examines the effect of these transient alterations on the efficacy of BPD-MA-mediated PDT in a subcutaneous (s.c.) rat prostate cancer model.

In an earlier study, the penetration of pulsed 690 nm light was measured in a tissue-simulating system in which the absorption, scattering and photosensitizer levels could be set arbitrarily (Pogue et al, 1997; van Staveren et al, 1991). The results indicated that the depth of necrosis from light-activated sensitizer may be 1–5 mm greater with 10 ns pulsed-laser light in the range of 10 MW cm$^{-2}$, as compared to cw irradiation, depending upon the sensitizer and the tissue optical properties. These initial measurements (Pogue et al, 1997; Pogue et al, 1996) of our own and the previous studies of others (Cowled et al, 1984; McKenzie and Carruth, 1986; Ben-Hur et al, 1987; Ferrario et al, 1991; Shikowitz, 1992; Panjehpour et al, 1993; Rausch et al, 1993; Okunaka et al, 1992) mentioned above were the motivation for these in vivo measurements of light penetration, and comparisons of depth of necrosis in a rat s.c. tumour model, using BPD-MA as the sensitizer and comparing 10 ns pulses with cw irradiation.

### MATERIALS AND METHODS

### Photosensitizer

Liposomal BPD-MA was a gift of QLT Phototherapeutics Inc. (Vancouver, BC, Canada) and was freshly reconstituted in 5% dextrose solution at a concentration of 1.0 mg ml$^{-1}$. The rats were injected intravenously (i.v.) in the tail with 5 mg kg$^{-1}$ photosensitizer for the optical transmission studies, and 2 mg kg$^{-1}$ for the PDT depth of necrosis measurements.

### Cell lines

The tumour cell line R-3327 MatLyLu was a generous gift of Dr W Heston (Memorial Sloan-Kettering Cancer Center, New York, NY, USA) and was derived from a rat prostate cancer, which frequently forms metastases to the lung and lymph nodes. These cells were grown in RPMI-1640 with L-glutamine (Mediatech, Washington, DC, USA) and supplemented with 10% fetal bovine serum (FBS; Gibco, Grand Island, NY, USA) and penicillin–streptomycin (100 units ml$^{-1}$ penicillin G, 100 μg ml$^{-1}$ streptomycin, Sigma, St Louis, MO, USA). Cells were incubated at 37°C in an atmosphere of 5% carbon dioxide and were passaged every 4–5 days.

### Animals

All animal procedures were approved by the Institutional Subcommittee on Research for Animal Care. Experiments were carried out on male Copenhagen rats (Sprague-Dawley). The animals were anaesthetized with a ketamine cocktail (ketamine, xylazine and atropin) after slow inhalation of metofane. After anaesthesia, $5 \times 10^5$ cells in 0.1 ml of PBS were injected s.c. into the skin on the hind leg of each animal. At 2–3 weeks after injection, when tumours grew to approximately 10 mm in diameter, PDT experiments were carried out.

### Irradiation sources

The cw light at 690 nm produced from an argon ion pumped dye laser (CR-599, Coherent) was delivered to the tumour surface through a 500 μm core fibre optic and a flat top beam profile was used for all irradiations with collimation provided by a lens system. The 690-nm pulsed light was produced from a Nd/YAG pumped Optical Parametric Oscillator (MOPO-700, SpectraPhysics), operating at 10 Hz pulse repetition frequency, and a beam diameter of 7 mm. The beam was cropped with an aperture at 4 mm diameter, and expanded to 16 mm diameter with a lens system, so the radial variation in intensity was less than 20%. All power measurements were taken with a calibrated power meter (Scientec model 365, CO).

### Tissue simulation

The penetration of light in a scattering medium with photosensitizer was examined as a function of laser pulse energy to establish the magnitude of the effects, and to confirm theoretical predictions. The attenuation due to BPD-MA absorption was examined first in a well-characterized scattering medium that simulates the scattering pattern within tissue, to avoid the complexities associated with animal tissues. A lipid emulsion, Intralipid (Clintec, Mississauga, Canada), was used for these measurements since it has well-characterized scattering and absorption coefficients (Aveline et al, 1994). Samples of 2% Intralipid in distilled water were used with and without 10 μg ml$^{-1}$ BPD-MA for measurements of reflectance and transmittance as a function of pulse energy.

### Transient absorbance measurement

The diffuse reflectance and diffuse transmittance were measured for both scattering solutions and tissue slices as a function of laser pulse energy. The experimental setup was arranged so that a single laser pulse could be used for each transmittance or reflectance measurement. The incident power was measured by splitting off 4% of the incident laser energy into a radiometer (Laser Precision RJ7610) positioned before the sample. The reflectance and transmittance were measured from thin samples with a silicon photodiode (Thor Labs FDS010, Newton, NJ, USA) at a distance of 50 cm from the sample. The samples were held in 1-mm-thick quartz cuvettes that had a width of 20 mm, so that edge effects in transmittance were negligible. Transmittance and reflectance measurements were made on Intralipid solutions as well as with tissue slices of muscle and liver taken from rats immediately after sacrificing. BPD-MA was injected at 5 mg kg$^{-1}$ into the tail vein of these rats, 1 h before sacrificing, and control animal tissue was taken from animals that had not been injected with BPD-MA but were injected with vehicle alone.

### Computer simulation

A computer program was used to estimate the population of photosensitizer molecules within a diffusely scattering medium, which is irradiated with pulsed or cw laser light. This program has been described in detail previously (Pogue et al, 1996), and is a standard finite difference calculation of both the light propagation by diffusion theory, and the photochemical rate equations of the sensitizer molecules at all points within the medium. The program can estimate the relative reflectance and transmittance from a sample based upon the input laser pulse energy and time length, the absorption and scattering coefficients of the background medium, the concentration of the photosensitizer along with its associated photophysical properties.

### Photodynamic treatment

BPD-MA was injected into the tail vein of the rats at 2 mg kg$^{-1}$, 1 h prior to irradiation. For irradiation, the skin was sterilized with ethanol and carefully pulled back from the surface of the tumour so that the light was directly incident upon the tumour surface. All rat tumours were exposed to 50 J cm$^{-2}$ total incident light dose, and were either treated with the pulsed or the cw light. For cw light, the irradiance was 200 mW cm$^{-2}$, while for pulsed light, peak irradiances used were 1.25 and 2.25 MW cm$^{-2}$ with these corresponding to average irradiances of 125 and 225 mW cm$^{-2}$, respectively, since the pulse length used was approximately 10 ns. This corresponds to a pulse fluence of 12.5–22.5 mJ cm$^{-2}$ per pulse. After irradiation the tumour skin was sutured closed and rats were sacrificed at 48 h post-treatment by inhalation with metofane and the tumour tissue was removed for histology.

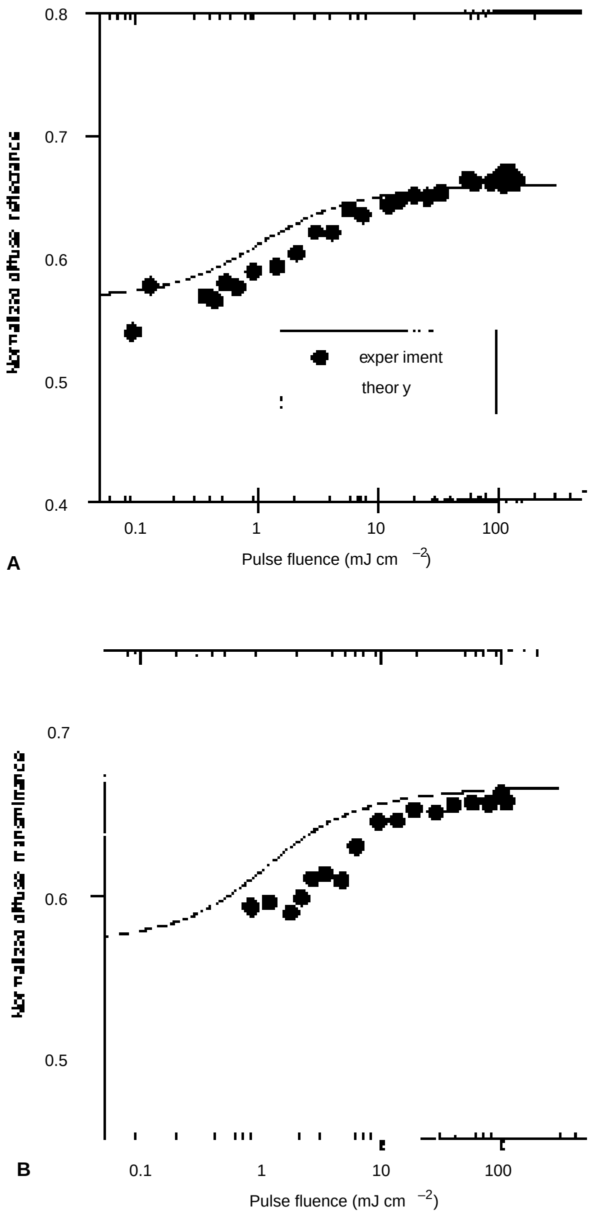

**Figure 1** **(A)** Diffuse reflectance measured from 1-mm-thick samples of 2% Intralipid with 10 μg ml$^{-1}$ BPD-MA, normalized by the reflectance measured without BPD-MA. Measurements were taken with a single laser pulse for each point. The solid lines are data from a computer simulation of the experiment, where the concentration of BPD-MA was used as the only free fitting parameter, other parameters were from measured values from reference (37). **(B)** Diffuse transmittance measured for the same conditions as described in **(A)**

### Histology

Three sections, at 2-mm intervals, were taken perpendicularly with respect to the superficial surface from each of the individual excised tumour specimens. These tissues were then preserved in 10% aqueous formalin, processed and paraffin embedded by standard methods. The histology slides were all stained with haematoxylin and eosin. The depth of the treatment effect was calculated using ocular micrometer attached to an Olympus BH-2 microscope. In each slice, the depth of treated tissue was calculated as a average of three depth measurements on the same slide, performed

*© Cancer Research Campaign 1999*

by one investigator and a pathologist blinded to the nature of the treatments.

### RESULTS

### Tissue-simulating medium

Figure 1A shows the reflectance of light from the sample with BPD-MA, normalized by the reflectance from the sample without sensitizer (control sample). Similarly, Figure 1B is the transmittance for the BPD-MA sample, normalized by the identical measurements from the control sample. The values in Figure 1 are relative, but using these values, the absolute reflectance was also estimated by normalizing with the near 100% reflectance from a sample of $Ba_2SO_4$ powder (i.e. very high albedo sample). The absolute reflectance from the 2% Intralipid with BPD-MA corresponded to 0.35 and 0.41 for pulse energies of 0.1 and 100 mJ cm$^{-2}$ respectively. These reflectance measurements were compared to a table of reflectance and transmittance values from a Monte Carlo simulation (Wang and Jacques, 1992). Assuming that scattering coefficient and anisotropy coefficient were given by van Staveren et al (1991) as $\mu_s$ = 7.8 mm$^{-1}$ and g = 0.70, the absorption coefficient corresponding to these absolute reflectance values is 0.25 and 0.14 mm$^{-1}$, respectively, for the pulse energies of 0.1 and 100 mJ cm$^{-2}$.

The solid lines on Figure 1A and 1B are the results of a finite difference calculation of light propagation through a diffusive medium using both the diffusion theory of light propagation in a highly scattering medium and the intensity-dependent population changes in the photosensitizer molecules. In this case, the concentration of the photosensitizer was adjusted to fit the data, but all other photophysical properties were fixed to those measured by Aveline et al (1994). It is interesting to note from the theoretical curve in Figure 1 that the regions of zero slope at low and high pulse energies correspond to the majority of the photosensitizer molecules in the ground and excited states respectively.

### Tissue reflectance/transmittance

The diffuse reflectance and transmittance were also measured using excised tissue slices from rats to observe the same type of changes in absorption with pulse energy. The reflectance versus pulse energy was measured for liver tissue and muscle tissue, both with and without BPD-MA; the results are plotted in Figure 2A. The transmittance values for the same tissues are plotted in Figure 2B. The transmittance and reflectance values were constant before and after the experiment suggesting that there was no significant permanent photobleaching of either the tissue components or BPD-MA. The lines on the Figures are simple linear regression fits to a straight line. For reflectance, the slopes for muscle with and without BPD-MA were 1.4 (± 0.4) × 10$^{-4}$ and 1.1 (± 0.4) × 10$^{-4}$, respectively, which are not statistically different, and the slopes for liver with and without BPD-MA were 9.5 (± 2.7) × 10$^{-5}$ and 9.4 (± 2.0) × 10$^{-5}$, respectively, which are also not statistically significant. The slopes of the transmittance versus pulse energy for muscle with and without BPD-MA are 2.4 (± 0.4) × 10$^{-5}$ and 0.4 (± 0.4) × 10$^{-5}$, respectively, which are significantly different at the $P < 0.0001$ level. The slopes of the transmittance through liver versus pulse energy with and without BPD-MA are 4.3 (1.2) × 10$^{-6}$ and 8.7 (2.7) × 10$^{-8}$, which are significantly different at the $P < 0.002$ level.

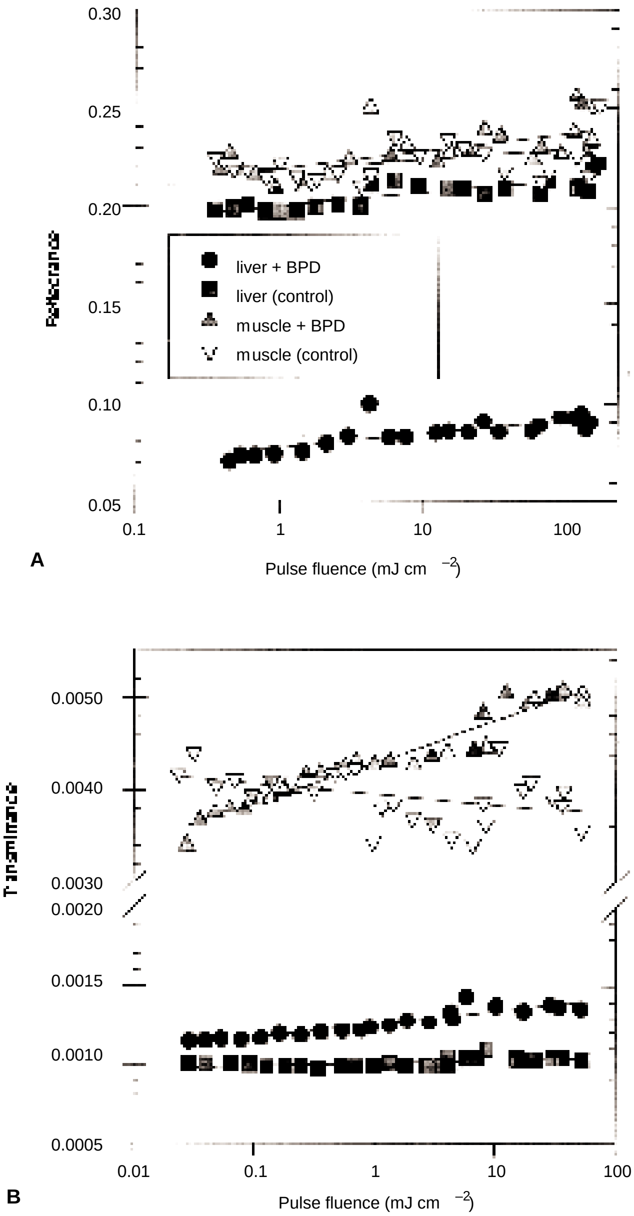

**Figure 2** **(A)** Relative reflectance from liver and muscle sections, both with and without BPD-MA present. BPD-MA was injected at a dose of 5 mg kg$^{-1}$ into rats, 1 h prior to taking tissue samples. **(B)** Relative transmittance from the same 1-mm-thick samples as in **(A)**

Previous studies of biodistribution of liposomal BPD-MA in mice (Richter et al, 1993) showed that the uptake of the drug in liver is 60–80% of the injected dose, and in muscle is approximately 1.5% of the injected dose. These relative uptake numbers explain why the reflectance of the liver is reduced from 0.20 normally to 0.07 with BPD-MA, in Figure 2A, while the reflectance from muscle does not change significantly with the addition of BPD-MA. The approximate transport scattering and absorption coefficients for rat liver and muscle at 690 nm are $\mu_s'$ (liver) = 1.0 mm$^{-1}$, $\mu_a$ (liver) = 0.4 mm$^{-1}$ (39), and $\mu_s'$ muscle = 0.5 mm$^{-1}$, $\mu_a$ (muscle) = 0.01 mm$^{-1}$ (Henderson et al, 1985). Using these values, the absorption due to the photosensitizer based upon Monte Carlo calculations of the diffuse reflectance in liver is

© Cancer Research Campaign 1999 

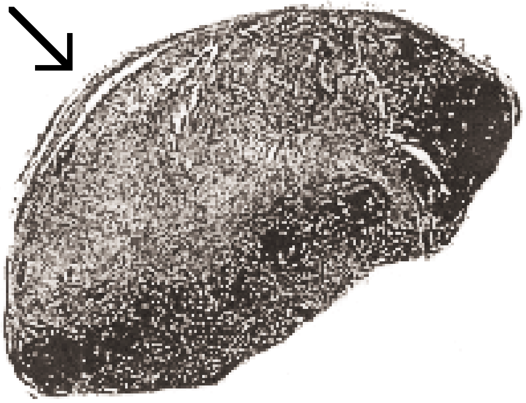

A

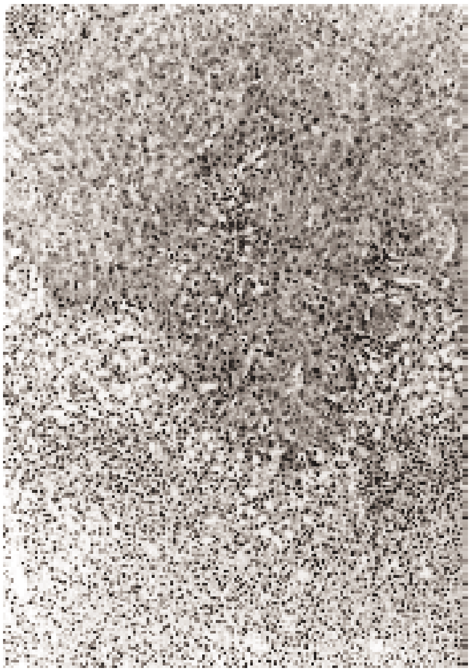

B

**Figure 3** **(A)** Overview of an entire slide of a histologic section of a tumour irradiated with pulsed light from the top (arrow). **(B)** Section of the same histologic slice of a tumour irradiated with pulsed laser light showing the demarcation between viable and necrotic zones. In both cw and pulsed irradiation cases light was delivered from the top and the treatment affected tissue showed necrotic cells (light grey), while the viable tissue with healthy cells with large nuclei is dark grey approximately 0.5 mm$^{-1}$, corresponding to a concentration of 60 μM of BPD-MA.

### Depth of treatment

Once the reflectance and transmittance behaviour in the presence and absence of BPD-MA had been established in tissue-simulating media and in tissue slices ex vivo, the average depth of necrosis for pulsed and cw light was measured in vivo in the s.c. MatLyLu tumours grown in Copenhagen rats. The treatment, as described above, was completed for a cw dose-rate of 200 mW cm$^{-2}$ and for pulsed dose rates of 125 and 225 mW cm$^{-2}$, using 7–8 rats for each condition. A typical histologic slice is shown in Figure 3 for both pulsed irradiation. The histologic appearance was similar for cw

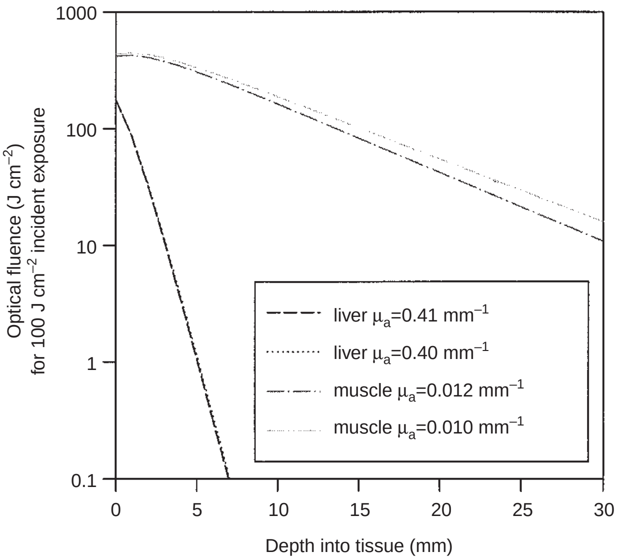

**Figure 4** The fluence distribution as a function of depth into a semi-infinite medium, for optical properties typical of liver and muscle, both with and without the absorption due to photosensitizer (for liver $\mu_a$ = 0.4 mm$^{-1}$, $\mu_s'$ = 1.0 mm$^{-1}$, and for muscle $\mu_a$ = 0.01, $\mu_s'$ = 0.5 mm$^{-1}$)

irradiation. The dead region of the tumour is clearly visible as light grey, while the viable region of the tissue was stained darker. Histologic inspection of the treated tumours revealed irregular boundaries between viable and necrotic tissue, with the viable tissue surrounding intact blood vessels. In fields of widespread cellular death, the endothelial cells from the blood vessels had also degenerated. These patterns are consistent with necrosis resulting from initial traumatization of the more delicate blood vessels, followed by secondary ischaemia and infarction, rather than direct photochemical effects on the malignant cells although no attempt was made to distinguish between different mechanisms of tumour destruction.

The summary of data from all treated tumours is listed in Table 1, showing average depth of treatment-affected cells as measured from three histology slides per tumour (21–24 samples per point). The overall averages for treatment depth are 8.7 ± 2.5 mm, 7.7 ± 2.3 mm and 8.5 ± 1.8 mm for the three irradiation conditions of cw (200 mW cm$^{-2}$), pulsed low irradiance (125 mW cm$^{-2}$) and pulsed high irradiance (225 mW cm$^{-2}$) respectively. These three depth values are not statistically different from each other.

### DISCUSSION

The aim of this paper was to examine transient absorption changes in tissue with high-intensity pulsed laser light and to determine if at moderate photosensitizer doses there was any significant benefit for treatment outcome from PDT when pulsed irradiations with high-intensity light sources were used. Our previous study in tissue-simulating phantoms indicated that pulsed laser light at high intensities could cause a transient change in the absorption of the sensitizer by exciting large fractions of the molecules to the excited states within the time of the laser pulse (Pogue et al, 1997). For sensitizers which have lower extinction coefficients in the upper singlet- and triplet-states than in the ground-state, such population changes are observed as a decrease in the absorption of the sensitizer. The transient decrease in absorption then allows a higher

 © Cancer Research Campaign 1999

fraction of the light to be transmitted to the deeper tissue layers, and the magnitude of this effect can be estimated from the transmission measurements from thin tissue slices. The program used here to calculate the reflectance and transmittance shows that the transient change in absorption can be predicted based upon the theoretical extinction coefficients of the medium. The data show that the net benefit to PDT depth of treatment is small, but it depends upon the optical properties of the tissues irradiated, the absorption due to the photosensitizer and the peak pulse irradiance.

From Figure 1A, the absorption coefficient values of the medium can be recovered by comparison to the Monte Carlo table of values assuming that the scattering coefficient is given by the published value (van Staveren et al, 1991). The absorption coefficient for the ground-state and the triplet-state are 0.25 $mm^{-1}$ and 0.14 $mm^{-1}$, respectively, using the low-pulse fluence region of reflectance for the ground-state and the high-pulse fluence for the triplet-state. The ratio of these two absorption coefficients is equal to the ratio of the theoretical extinction coefficients for BPD-MA ground- and triplet-states ($\varepsilon_{Ground}$ = 34 000 $M^{-1}$ $cm^{-1}$ and $\varepsilon_{Triplet}$ = 20 000 $M^{-1}$ $cm^{-1}$) (35). Measurement of reflectance and transmittance from Intralipid with BPD-MA demonstrates that it is possible to completely convert this molecule to triplet-state within a scattering medium. This sigmoid-shaped curve of transmittance versus pulse fluence (Figure 1B) is also observed in non-scattering media, where the two parts of the curve with zero slope correspond to stable states of the molecule with differing extinction coefficients (Wilson et al, 1990). Usually, changes in transmittance such as these are recorded with two laser pulses, where the first excites the molecules and the second is the measurement; however, in this case the excitation and change can be observed with a single laser pulse. Below pulse fluences of 0.1 mJ $cm^{-2}$, the photosensitizer is predominantly in the ground-state, and so the attenuation of the pulse is determined by the product of the concentration and ground-state extinction coefficient of the photosensitizer. Above pulse fluences of 10 mJ $cm^{-2}$, most of the chemical is converted to the triplet-state, and the absorption is given by the product of the concentration and the triplet-state extinction coefficient of the photosensitizer.

The conversion of BPD-MA to excited states in the excised rat tissue was successfully measured in the same manner as with Intralipid solutions, from the reflectance and transmittance measurements as a function of pulse energy (Figure 2). In these experiments diffuse transmittance measurements indicate an absorption decrease with increasing pulse energy, while the reflectance intensity does not change with pulse energy presumably because the light has followed a longer average path through the tissue for transmittance than for the diffusely reflected light and is therefore more sensitive to absorption changes. The transmittance of pulsed light near pulse fluences of 30 mJ $cm^{-2}$ as compared to below 0.1 mJ $cm^{-2}$, is approximately 25% higher for liver, and 40% higher for muscle through 1-mm samples.

By comparing the transmittance change to calculated values of transmittance based upon Monte Carlo calculations, and assuming typical values of the optical interaction coefficients of muscle and liver (see Results section), the change in absorption coefficients were approximately $\Delta\mu_a$ (liver) = 0.01 $mm^{-1}$ and $\Delta\mu_a$ (muscle) = 0.002 $mm^{-1}$. These changes in absorption can be used to estimate the increase in depth of necrosis based upon the light penetration into the tissue. Figure 4 shows a theoretical prediction of the optical light fluence depth profile in an infinitely thick slab of tissue, where four sets of optical properties have been used in the calculation. The first two correspond to liver with drug and liver with drug that has been transiently bleached due to the laser pulse, with predicted intrinsic absorption of $\mu_a$ = 0.40 $mm^{-1}$ and scattering of $\mu_s'$ = 1.0 $mm^{-1}$. The second two show the same calculation with muscle tissue, where the background absorption is approximately $\mu_a$ = 0.01 $mm^{-1}$, and scattering is $\mu_s'$ = 0.5 $mm^{-1}$. For a given fluence, there is negligible increase in the depth for liver tissue, and between 0 and 3 mm increase for the muscle tissue. Based upon these diffusion theory calculations, the maximum increase in optical penetration with these changes in absorption is between 0 and 1.0 mm, in either liver or muscle as well as tumour tissue. These data would suggest only a modest increase with pulsed irradiation in the depth of necrosis at 5 mg $kg^{-1}$ injected photosensitizer dose. These effects would be expected to be further reduced at lower BPD-MA concentrations because of reduced shielding.

Consistent with the above data, the measurements of depth of necrosis results listed in Table 1, directly comparing cw to pulsed treatments, for similar average incident dose-rates show no statistically different change in the depth of treatment response for the 2 mg $kg^{-1}$ of BPD-MA dose used here. It is possible that increased depth of necrosis could be associated with high intensity pulsed laser PDT from physiologic effects such as localized hyperthermia generation (Henderson et al, 1985; Leunig et al, 1994), or oxygen depletion effects during the irradiation (Foster et al, 1991; Gibson et al, 1994; Hua et al, 1995). Since the same average dose was used for pulsed and cw light the level of heat production or oxygen consumption should have been comparable. At this point it is not obvious what the basis for the reported differences in PDT effects with pulsed and cw irradiation may have been (Okunaka et al, 1992; Rausch et al, 1993). It is possible that alternative photosensitizing pathways are initiated with pulsed light, such as radical species produced via the upper excited states of the photosensitizer. However, these radical species would be produced in greatest number in the upper surface tissue layers where the fluence is the highest, and so they are not likely to contribute to an increased depth of necrosis. There is also no obvious indication that there should be a difference with other sensitizers or tissue types based upon our calculations. The only obvious benefit of pulsed light is the transient change in absorption which would be most significant in tissues where the absorption due to the photosensitizer (i.e. extinction coefficient times the concentration) is much greater than the intrinsic absorption coefficient of the tissue, and when there is a lower extinction coefficient of the excited states as compared to the ground-state at the excitation wavelength. Although the BPD-MA concentrations used here were moderate, they were still higher than the typical concentrations used clinically. Since the current trend in PDT treatment protocols is to use lower photosensitizer doses, often below 1 mg $kg^{-1}$, this condition is unlikely to be experienced clinically except in highly translucent tissue types, so that shielding effects (and overcoming them with high-intensity pulsed irradiation) is unlikely to play an important role in PDT.

This study also demonstrates that transient population changes can be observed in tissue, and may be quantitatively estimated from changes in transmittance or reflectance. These changes could be observed with arbitrary accuracy using an optimized experimental setup, so that the triplet-state spectrum and triplet-state intersystem crossing rate can be measured in vivo with this type of method. These measurements could be of considerable use in PDT dosimetry as a diagnostic probe to determine the active fraction of

*© Cancer Research Campaign 1999*

the photosensitizer within the tissue (i.e. the fraction of photosensitizer that can be in the triplet-state). There may be significant differences in the active fraction between various photosensitizers in vivo because of where they localize and the extent of binding in the tumour tissue environment (Aveline et al, 1995). Such information could potentially be used to quantify PDT dosimetry and better compare different potential photosensitizers.

### ACKNOWLEDGEMENTS

This research has been funded by the DOD MFEL program contract # N00014-94-I-0927 (BWP, TM, TH). B Pogue and T Hasan would like to thank Drs Robert Redmond, Brian C Wilson, Lothar Lilge and Franz Hillenkamp for thoughtful discussions. The authors would like to acknowledge the generous gift of BPD-MA from QLT Phototherapeutics Inc. (Vancouver, British Columbia, Canada), and the loan of the Argon laser from Coherent Inc. (Palo Alto, CA, USA).

### REFERENCES

Andreoni A (1987) Two-step photoactivation of hematoporphyrin by excimer-pumped dye-laser pulses. *J Photochem Photobiol B Biol* **1**: 187–193

Andreonia A, Cubeddu R and Silvestrix D (1982) Two-step laser activation of hematoporphyrin derivative. *Chem Phys Lett* **88**: 37

Aveline B, Hasan T and Redmond RW (1994) Photophysical and photosensitizing properties of benzoporphyrin derivative monoacid ring A (BPD-MA). *Photochem Photobiol* **59**: 328–335

Aveline BM, Hasan T and Redmond RW (1995) The effects of aggregation, protein binding and cellular incorporation on the photophysical properties of benzoporphyrin derivative monoacid ring A (BPD-MA). *J Photochem Photobiol B Biol* **30**: 161–169

Ben-Hur E, Newman HC, Crane SW and Rosenthal I (1987) Pulsed versus continuous-wave 680 nm laser light in phtosensitization by chloroaluminium phthalocyanine tetrasulfonate. New Directions in Photodynamic Therapy. *Proc SPIE* **847**: 154–157

Bown SG, Tralau CJ, Smith PD, Akdemir D and Wieman TJ (1986) Photodynamic therapy with porphyrin and phthalocyanine sensitisation: quantitative studies in normal rat liver. *Br J Cancer* **54**: 43–52

Cowled PA, Grace JR and Forbes IJ (1984) Comparison of the efficacy of pulsed and continuous-wave red laser light in induction of photocytotoxicity by haematoporphyrin derivative. *Photochem Photobiol* **39**: 115–117

D'Hallewin MA and Baert L (1995) Long-term results of whole bladder wall photodynamic therapy for carcinoma in situ of the bladder. *Urol* **45**: 763–767

Farrell TJ, Wilson BC, Patterson MS and Chow R (1991) The dependence of photodynamic threshold dose on treatment parameters in normal rat liver in vivo. *Proc SPIE* **1426**: 146–155

Ferrario A, Rucker N, Ryter SW, Doiron DR and Gomer CJ (1991) Direct comparison of in-vitro and in-vivo Photofrin-II mediated photosensitization using a pulsed KTP pumped dye laser and a continuous wave argon ion pumped dye laser. *Lasers Surg Med*, **11**: 404–410

Foster TH, Murant RS, Bryant RG, Knox RS, Gibson SL and Hilf R (1991) Oxygen consumption and diffusion effects in photodynamic therapy. *Rad Res* **126**: 296–303

Gibson SL, Foster TH, Feins RH, Raubertas RF, Fallon MA and Hilf R (1994) Effects of photodynamic therapy on xenografts of human mesothelioma and rat mammary-carcinoma in nude-mice. *Br J Cancer* **69**: 473–481

Gomer CJ (1991) Preclinical examination of first and second generation photosensitizers used in photodynamic therapy [Review]. *Photochem Photobiol* **54**: 1093–1107

Hasan T and Parrish JA (1996) Photodynamic therapy of cancer. In *Cancer Medicine*, 4th Edn, Vol. XIII, Holland JF, Frei E III, Bast RC Jr, Kufe DW, Morton DL and Weichselbaum RR (eds) pp. 739–751. Williams & Wilkins: Baltimore, MD

Henderson BW, Waldow SM, Potter WR and Dougherty TJ (1985) Interaction of photodynamic therapy and hyperthermia: tumor response and cell survival studies after treatment of mice in vivo. *Cancer Res* **45**: 6071–6077

Hua ZX, Gibson SL, Foster TH and Hilf R (1995) Effectiveness of delta-aminolevulinic acid-induced protoporphyrin as a photosensitizer for photodynamic therapy in-vivo. *Cancer Res* **55**: 1723–1731

Jocham D, Baumgartner R, Stepp H and Unsold E (1990) Clinical experience with the integral photodynamic therapy of bladder carcinoma. *J Photochem Photobiol B Biol* **6**: 183–187

Keir WF, Land EJ, MacLennan AH, McGarvey DJ and Truscott TG (1987) Pulsed radiation studies of photodynamic sensitizers: the nature of DHE. *Photochem Photobiol* **46**: 587–589

Leunig M, Leunig A, Lankes P and Goetz AE (1994) Evaluation of photodynamic therapy-induced heating of hamster melanoma and its effect on local tumour eradication. *Inter J Hypertherm* **10**: 297–306

Lui H and Anderson RR (1993) Photodynamic therapy in dermatology: recent developments [Review]. *Derm Clinics* **11**: 1–13

McKenzie AL and Carruth JAS (1986) A comparison of Gold-vapor and dye lasers for photodynamic therapy. *Lasers Med Sci* **1**: 117–120

Moghissi K, Dixon K, Hudson E and Stringer M (1995) Photodynamic therapy of esophageal cancer. *Laser Med Sci* **10**: 67–71

Muller PJ and Wilson BC (1990) Photodynamic therapy of malignant brain tumours. *Canadian J Neurol Sci* **17**: 193–198

Narayan S and Sivak MV Jr (1994) Palliation of esophageal carcinoma. Laser and photodynamic therapy [Review]. *Chest Surg Clinics North Am* **4**: 347–367

Okunaka T, Kato H, Konaka C, Sakai H, Kawabe H and Aizawa K (1992) A comparison between argon-dye and excimer-dye laser for photodynamic effect in transplanted mouse tumor. *Jap J Cancer Res*, **83**: 226–231

Panjehpour M, Overholt BF, Denovo RC, Petersen MG and Sneed RE (1993) Comparative study between pulsed and continuous wave lasers for Photofrin photodynamic therapy. *Lasers Surg Med* **13**: 296–304

Pass HI (1993) Photodynamic therapy in oncology: mechanisms and clinical use. [Review] *J Nat lCancer Inst* **85**: 443–456

Patterson MS and Wilson BC (1994) A theoretical study of the influence of photosensitizer photobleaching on depth of necrosis in photodynamic therapy. *Proc SPIE* 2133

Pe MB, Ikeda H and Inokuchi T (1994) Tumour destruction and proliferation kinetics following periodic, low power light, haematoporphyrin oligomers mediated photodynamic therapy in the mouse tongue. *Oral Oncol Euro. J Cancer: Part B*, **30B**: 174–178

Pogue BW, Redmond RW and Hasant T (1996) A study of dosimetry for pulsed-laser photodynamic therapy. *Proc SPIE* **2681**: 130–139

Pogue BW, Lilge L, Patterson MS, Wilson BC and Hasant T (1997) The absorbed photodynamic dose examined from pulsed and cw light using tissue-simulating dosimeters. *Appl Opt* **36**: 7257–7269

Rausch PC, Rolfs F, Winkler MR, Kottysch A, Schauer A and Steiner W (1993) Pulsed versus continuous-wave excitation mechanisms in photodynamic therapy of differently graded squamous-cell carcinomas in tumor-implanted nude-mice. *Eur Arch Oto Rhino Laryngol* **250**: 82–87

Richter AM, Waterfield E, Jain AK, Canaan AJ, Allison BA and Levy JG (1993) Liposomal delivery of a photosensitizer, benzoporphyrin derivative monoacid ring A (BPD), to tumor tissue in a mouse tumor model. *Photochem Photobiol* **57**: 1000–1006

Rosenberg SJ and Williams RD (1986) Photodynamic therapy of bladder carcinoma. *Urol Clinics North Am* **13**: 435–444

Shea CR, Hefetz Y, Gilles R, Wimberly J, Dalickas G and Hasan T (1990) Mechanistic investigation of doxycycline photosensitization by picosecond-pulsed and continuous wave laser irradiation of cells in culture. *J Biol Chem* **265**: 5977–5982

Shikowitz MJ (1992) Comparison of pulsed and continuous wave light in photodynamic therapy of papillomas: an experimental study. *Laryngoscope* **102**: 300–310

Smith G, McGimpsey WG, Lynch MC, Kochevar IE and Redmond RW (1994) An efficient oxygen-independent two-photon photosensitization mechanism. *Photochem Photobiol* **59**: 135–139

Stiel H, Teuchner K, Leupold D, Oberlander S, Ehlert J and Jahnke R (1991) Computer aided laser-spectroscopic characterization and handling of molecular excited states. In *Intelligent Instruments and Computers*, pp. 79–88. Elsevier: Amsterdam

Stiel H, Marlow I and Roeder B (1993) Photophysical properties of the photosensitizer pheophorbide a studied at high photon flux densities. *J Photochem Photobiol B Biol* **17**: 181–186

Svaasand LO, Gomer CJ and Morinelli E (1990) On the physical rationale of photodynamic therapy. *SPIE Inst Series*, **Vol. IS 6**: 233–248

Van Staveren HJ, Moes CJM, van Marle J, Prahl SA and van Gemert MJC (1991) Light scattering in Intralipid-10% in the wavelength range of 400–1100 nm. *Appl Opt* **30**: 4507–4514

 © Cancer Research Campaign 1999

Wang L and Jacques S (1992) Monte Carlo modeling of light transport in multi-layered tissues in standard C (from FTP site for MD Anderson Cancer Center)

Wilson BC, Patterson MS and Burns DM (1986) Effect of photosensitizer concentration in tissue on the penetration depth of photoactivating light. *Lasers Med Sci* **1**: 235–244

Wilson BC, Farrell TJ and Patterson MS (1990) An optical fiber-based diffuse reflectance spectrometer for non-invasive investigation of photodynamic sensitizers in vivo. *Future Directions and Applications in PDT*, SPIE Inst. Series, **Vol. 6**: 219–232

Wilson BD, Mang TS, Stoll H, Jones C, Cooper M and Dougherty TJ (1992) Photodynamic therapy for the treatment of basal cell carcinoma. *Arch Derm* **128**: 1597–1601

*© Cancer Research Campaign 1999*
